# Two Distinct Categories of Focal Deletions in Cancer Genomes

**DOI:** 10.1371/journal.pone.0066264

**Published:** 2013-06-21

**Authors:** Megha Rajaram, Jianping Zhang, Tim Wang, Jinyu Li, Cem Kuscu, Huan Qi, Mamoru Kato, Vladimir Grubor, Robert J. Weil, Aslaug Helland, Anne-Lise Borrenson-Dale, Kathleen R. Cho, Douglas A. Levine, Alan N. Houghton, Jedd D. Wolchok, Lois Myeroff, Sanford D. Markowitz, Scott W. Lowe, Michael Zhang, Alex Krasnitz, Robert Lucito, David Mu, R. Scott Powers

**Affiliations:** 1 Cancer Genome Center, Cold Spring Harbor Laboratory, Woodbury, New York, United States of America; 2 Department of Neurosurgery, Cleveland Clinic, Cleveland, Ohio, United States of America; 3 Department of Genetics, The Norwegian Radium Hospital, Oslo, Norway; 4 Departments of Internal Medicine and Pathology, University of Michigan, Ann Arbor, Michigan, United States of America; 5 Departments of Medicine and Surgery, Memorial Sloan-Kettering Cancer Center, New York, New York, United States of America; 6 Department of Medicine and Ireland Cancer Center, Case Western Reserve University and University Hospitals of Cleveland, Cleveland, Ohio, United States of America; University of North Carolina School of Medicine, United States of America

## Abstract

One of the key questions about genomic alterations in cancer is whether they are functional in the sense of contributing to the selective advantage of tumor cells. The frequency with which an alteration occurs might reflect its ability to increase cancer cell growth, or alternatively, enhanced instability of a locus may increase the frequency with which it is found to be aberrant in tumors, regardless of oncogenic impact. Here we’ve addressed this on a genome-wide scale for cancer-associated focal deletions, which are known to pinpoint both tumor suppressor genes (tumor suppressors) and unstable loci. Based on DNA copy number analysis of over one-thousand human cancers representing ten different tumor types, we observed five loci with focal deletion frequencies above 5%, including the *A2BP1* gene at 16p13.3 and the *MACROD2* gene at 20p12.1. However, neither RNA expression nor functional studies support a tumor suppressor role for either gene. Further analyses suggest instead that these are sites of increased genomic instability and that they resemble common fragile sites (CFS). Genome-wide analysis revealed properties of CFS-like recurrent deletions that distinguish them from deletions affecting tumor suppressor genes, including their isolation at specific loci away from other genomic deletion sites, a considerably smaller deletion size, and dispersal throughout the affected locus rather than assembly at a common site of overlap. Additionally, CFS-like deletions have less impact on gene expression and are enriched in cell lines compared to primary tumors. We show that loci affected by CFS-like deletions are often distinct from known common fragile sites. Indeed, we find that each tumor tissue type has its own spectrum of CFS-like deletions, and that colon cancers have many more CFS-like deletions than other tumor types. We present simple rules that can pinpoint focal deletions that are not CFS-like and more likely to affect functional tumor suppressors.

## Introduction

In human cancer it is generally the case that highly recurrent point mutations, such as those occurring in *KRAS* or *TP53*, contribute to the selective advantage of tumor cells. However the case is less clear with DNA copy number alterations, where some frequent alterations such as amplification of the *ERBB2/HER2* locus clearly provide a selective advantage, whereas others like the frequent deletions of DNA at the telomeric ends of chromosomes likely do not. This means that alteration frequency alone is not sufficient to determine whether or not a given DNA copy number alteration directly impacts oncogenicity. Nowhere has this been more difficult to tease out than for candidate tumor suppressor genes located within common fragile sites. Common fragile sites are found throughout the human genome and are prone to DNA breaks when the cell is exposed to partial replication stress [1], [2]. Cancer cells frequently show hemizygous or homozygous deletions at these loci and in addition there are often expression alterations of the underlying gene [3]. Tumor suppressor functions have been found for some of these genes, including *WWOX*, *FHIT*, and *PARK2*, and these functions include growth suppressive effects of restoring expression in deficient cell lines and loss-of-function mutations leading to enhancement of carcinogen-induced or genetically-engineered cancer in mice [3], [4], [5]. Other studies have made observations that do not support a tumor suppressor role for these genes, including the inability to detect inactivating point mutations [6], [7] and the frequent failure of deletions to affect underlying RNA or protein expression [7], [8], both of which are common features of other tumor suppressor genes.

Previously we discovered and validated 26 oncogenes by functionally screening sets of genes that are focally amplified in human cancer and similarly have validated 10 tumor suppressor genes that were found in focal deletions affecting liver cancer [9], [10], [11], [12], [13], [14], [15]. We began this study with the goal of validating two tumor suppressor gene candidates that were focally deleted at high frequency particularly in colorectal cancer, *A2BP1* and *MACROD2*. In contrast to the results obtained by screening focal deletions in liver cancer, we did not find that these genes were tumor suppressors, which prompted us to take a genome-wide look at the properties of focally deleted genes in a large dataset of DNA copy number alterations affecting over 1000 cancer samples. This led to the discovery of two classes of focal deletions in human cancer, one that resembles deletions affecting common fragile sites and the other that resembles deletions affecting *CDKN2A/B*. Since then, a genome-wide examination of small homozygous deletions in 270 human cancer cell lines has been reported that also finds two classes of focal deletions [16], and while our conclusions are largely similar to theirs, there are some important distinctions and nuances about the two classes and additional findings which are described below.

## Results

### Common Sites of Focal Deletions in Human Cancer

**Figure 1:**
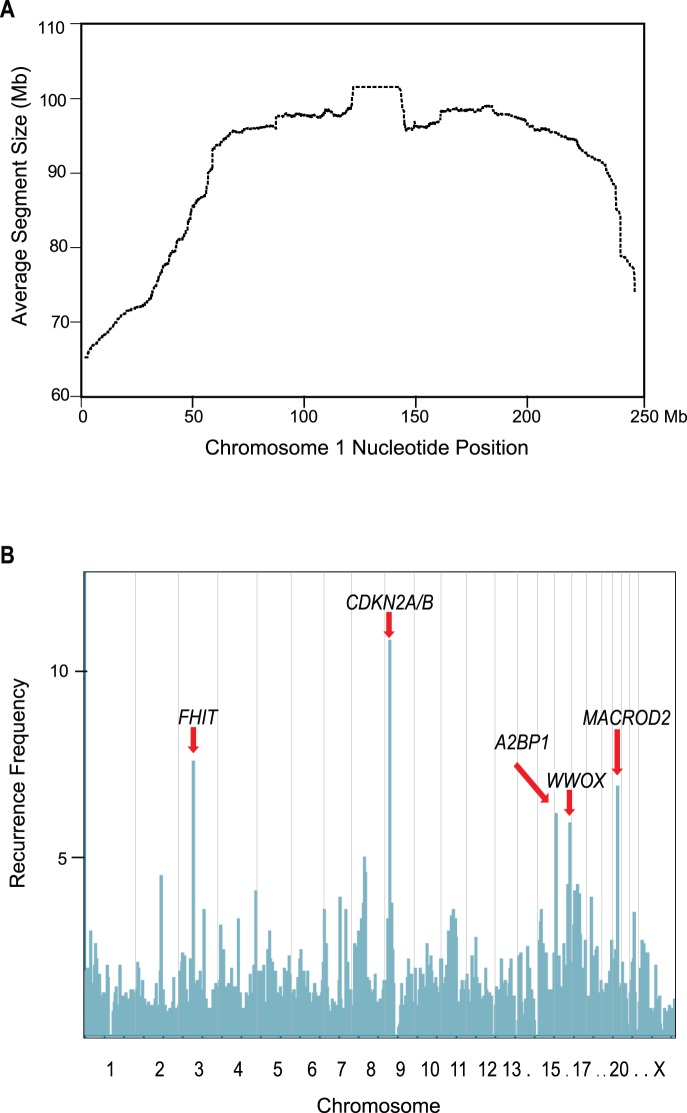
Sites in the genome of frequent cancer focal deletions. (A) Average DNA segment size as a function of position on chromosome 1 is displayed. (B) the 9,401 focal deletions that were not telomeric were binned into 2 Mb intervals and used to generate a frequency distribution across the genome. The most frequently affected genomic interval corresponded to the *CDKN2A/B* locus and the next six most frequently affected genomic intervals are indicated with red arrows and the relevant gene.

Our dataset was generated by array CGH analysis of 850 primary tumors and 304 cancer cell lines or xenografts of diverse tissue types including brain, breast, colon, liver, lung, ovarian, pancreatic, prostate, and skin (melanoma) (Table S1). Following data normalization and segmentation we detected a total of 10,835 focal deletions (<10 Mb) in these 1,154 samples (Table S2). The average size of deletions, both focal and large, is the shortest at telomeres as expected (Figure 1A, Figure S1 in File S1) and therefore a substantial percentage (13%) of focal deletions involved telomeric ends, particularly both ends of the X chromosome and the p arm of chromosome 4 (Table S3). Figure 1 shows the distribution of the other 87% of focal deletions across the genome, binned into 2-Mb intervals. There were five loci that showed focal deletion frequencies greater than 5% and they corresponded to the *CDKN2A/B*, *FHIT* and *WWOX* loci, known or suspected tumor suppressor genes, and the *MACROD2* and *A2BP1* loci (Figure 1B). Deletions affecting *A2BP1* (also known as *RBFOX1*) were very frequent in colorectal cancer (21%) but considerably less frequent or absent in other tumor types (1–4% in ovarian, liver and lung cancers and absent in breast, melanoma, prostate, and pancreatic cancers). Similarly, deletions affecting *MACROD2* were most frequent in colorectal cancer (17%) but considerably less frequent or absent in other tumor types (0.5–3% in breast, liver and lung cancers and absent in ovarian, melanoma, prostate, and pancreatic cancers). Frequent deletions affecting *A2BP1* and *MACROD2* in colon cancer have been observed by others [17], [18].

### Examination of *A2BP1* and *MACROD2* as Potential Tumor Suppressor Genes

**Figure 2:**
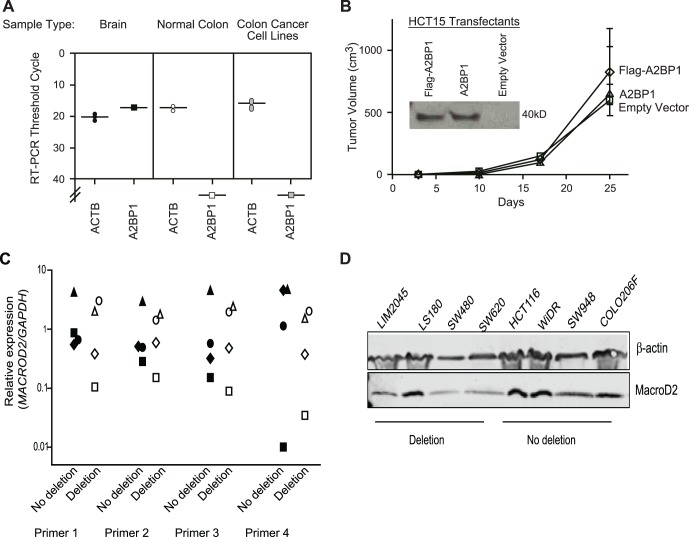
Expression and tumor suppressive properties of *A2BP1* and *MACROD2*. (A) Threshold PCR cycles for RT-PCR detection of *ACTB* (control) and *A2BP1* in two different samples of normal brain tissue, four different samples of normal colon tissue, and 19 colon cancer cell lines. Values below 40 indicate no signal detection. (B) The effect of forced ectopic expression of *A2BP1* on tumor formation of colon cancer cell line HCT-15, which harbors a 250-kb deletion within *A2BP1*. Detection of expression by immunoblotting using a polyclonal antibody that recognizes the A2BP1 protein [38] is shown in the insert. Lack of tumor suppressive effects were also observed for the colon cancer cell lines HCT-116 and SW480, both harboring deletions in *A2BP1*. (C) The expression of *MACROD2* in colon cancer cell lines as determined by TaqMan RT-PCR using four different probes (three to coding sequences and one to a 3′ UTR, all unaffected by deletions), comparing cell lines that harbor deletions within the *MACROD2* gene to ones that do not. Relative expression was calculated by the ΔC_T_ method using *GADPH* expression levels as the reference. (D) The expression of *MACROD2* in colon cancer cell lines as determined by immunoblotting using an antibody to MacroD2.

We examined the effects of *A2BP1* and *MACROD2* deletions on underlying gene expression in colon cancers and normal colon tissues. RNA expression of *A2BP1* could not be detected by real-time RT-PCR in any of the normal colon tissues, tumors, or cancer cell lines that we examined (Figure 2A). To help confirm this negative result, we designed three additional probes for real-time RT-PCR and standard RT-PCR, but in each case failed to detect *A2BP1* in colon samples, despite being able to readily detect its expression in the brain (Figure 2A). These results are consistent with a prior report that expression of *A2BP1/RBFOX1*, which encodes an alternative splicing factor, is restricted to the heart, muscle, and brain [19]. Although we cannot rule out very low-level but physiologically relevant expression of *A2BP1* in colon samples, we did not observe any growth suppressive or tumor suppressive effects of expressing *A2BP1* in colon cancer cell lines harboring deletions (Figure 2B). Additionally, although *MACROD2* is expressed in colon cancer cells, deletions had no effect on expression as measured by quantitative RT-PCR using four different probes including three within coding sequences and one in the 3′ untranslated region. Nor did deletions have any appreciable effect on expression of MacroD2 protein (Figure 2C). Although seemingly paradoxical, these deletions all occurred within introns of *MACROD2* and therefore would not necessarily be expected to affect expression.

### Patterns of Deletions Affecting *A2BP1, MACROD2, CDKN2A/B*, and *PTEN*

**Figure 3:**
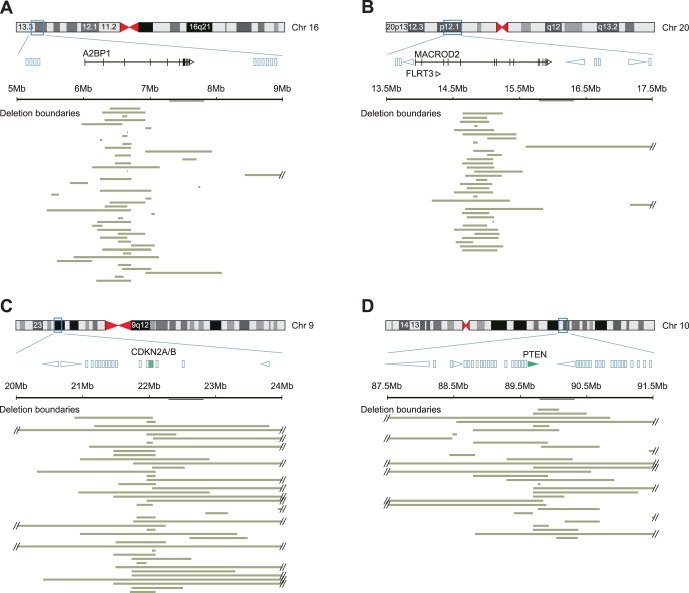
The boundaries of focal deletions that affect the *A2BP1*, *MACROD2*, *CDKN2A/B*, and *PTEN* genes. Each of the four panels shows (top) an ideogram of the chromosome on which the featured gene is located and (below) an expanded view of a 4-Mb region centered on the featured gene, showing other genes in the area (location based on the UCSC Genome Browser). If the gene is large enough, the exon-intron structure and/or transcriptional direction of the gene are indicated. For most genes, particularly in panels C and D, the genes are shown as rectangles due to their smaller size. Shown below each expanded region are horizontal bars indicating the extent and boundaries of individual deletions in colon tumors (Panels A and B; *A2BP1* and *MACROD2*, respectively), lung tumors (Panel C; *CDKN2A/B*), or multiple tumor types (Panel D; *PTEN*). The featured genes in Panels C and D are highlighted in green.

We noted several differences in the types and patterns of deletions affecting *A2BP1* and *MACROD2* loci when compared to focal deletions affecting *CDKN2A/B* and *PTEN* loci (Figure 3). By examining all focal deletions (<10 Mb) that spanned a four Mb locus centered on the target gene, we found that deletions affecting the *A2BP1* and *MACROD2* loci were on average smaller (0.6 Mb vs. 1.6 Mb, p = 1.5e-04). Additionally, the deletions affecting the *A2BP1* and *MACROD2* loci were more separated and the majority did not converge upon a single common site of overlap, in contrast to *CDKN2A/B* and *PTEN* loci (Figure 3). We measured the degree to which each deletion was separate (non-overlapping) from other deletions (“Deletion Separation”, see Materials and Methods) and found that there was a significant difference between the *A2BP1* and *MACROD2* loci and the *CDKN2A/B* and *PTEN* loci (0.4 vs. 0.16, p = 0.03).

**Figure 4:**
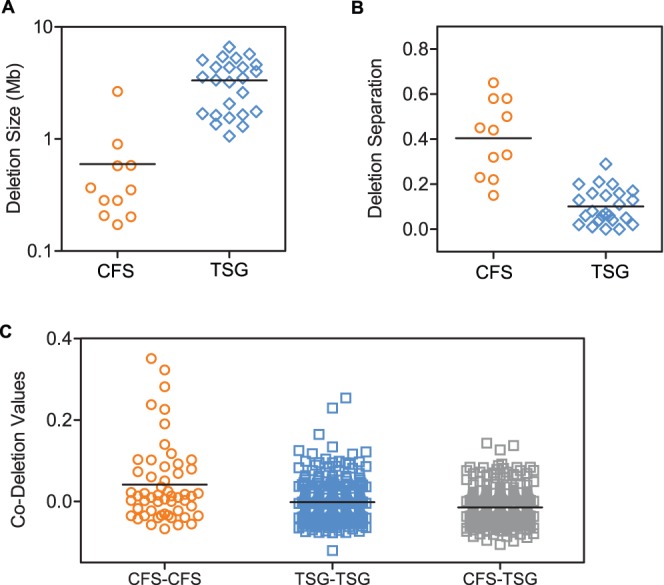
Deletion size and deletion separation can distinguish common fragile sites from tumor suppressor genes. (A) Box and whisker plots for “Deletion Size,” which measures the median size of deletions within a 2-Mb window centered on the gene, common fragile site genes (orange) and tumor suppressor genes (blue) (B) Box and whisker plots for “Deletion Separation,” which measures the separation (non-overlapping) of deletions within a 2-Mb window centered on the gene, (C) The co-deletion tendency of common fragile site genes (orange) and tumor suppressor genes (blue) relative to the co-deletion of genes of different classes (gray).

We then wanted to determine if these two distinctions between deletions affecting *A2BP1* and *MACROD2* loci on the one hand and *CDKN2A/B* and *PTEN* loci on the other hand held true when comparing deletions affecting known common fragile sites and recessive tumor suppressor genes from the Cancer Gene Census [20], [21]. In our dataset, there were 11 common fragile sites and 24 recessive tumor suppressor genes that contained a sufficient sample size of deletions (>14) for this statistical analysis (Table S4). Similar to what we observed above, in this larger set of loci the average deletion size affecting common fragile sites was significantly smaller than those affecting recessive tumor suppressors (0.6 Mb vs. 3.3 Mb, p = 9e-06, Figure 4A). Likewise, the “Deletion Separation” metric was significantly greater in common fragile sites than it was in recessive tumor suppressor genes (Figure 4B). This latter result suggests that deletions affecting these two groups arise by different mechanisms. Deletions in common fragile site genes can be induced by replicative stress and subsequent DNA damage and repair [22], deletions affecting *CDKN2A/B* have been suggested to arise from aberrant recombination or DNA repair by nonhomologous end-joining [23]. In support of the idea that these two types of deletions arise by separate mechanisms, we found that the frequency of co-occurrence of deletions in different common fragile site genes and co-occurrence of deletions in different tumor suppressor genes was greater than that of co-deletion of CFS and tumor suppressor genes (Figure 4C).

### Additional Properties that Distinguish Deletions Affecting Common Fragile Sites

**Figure 5:**
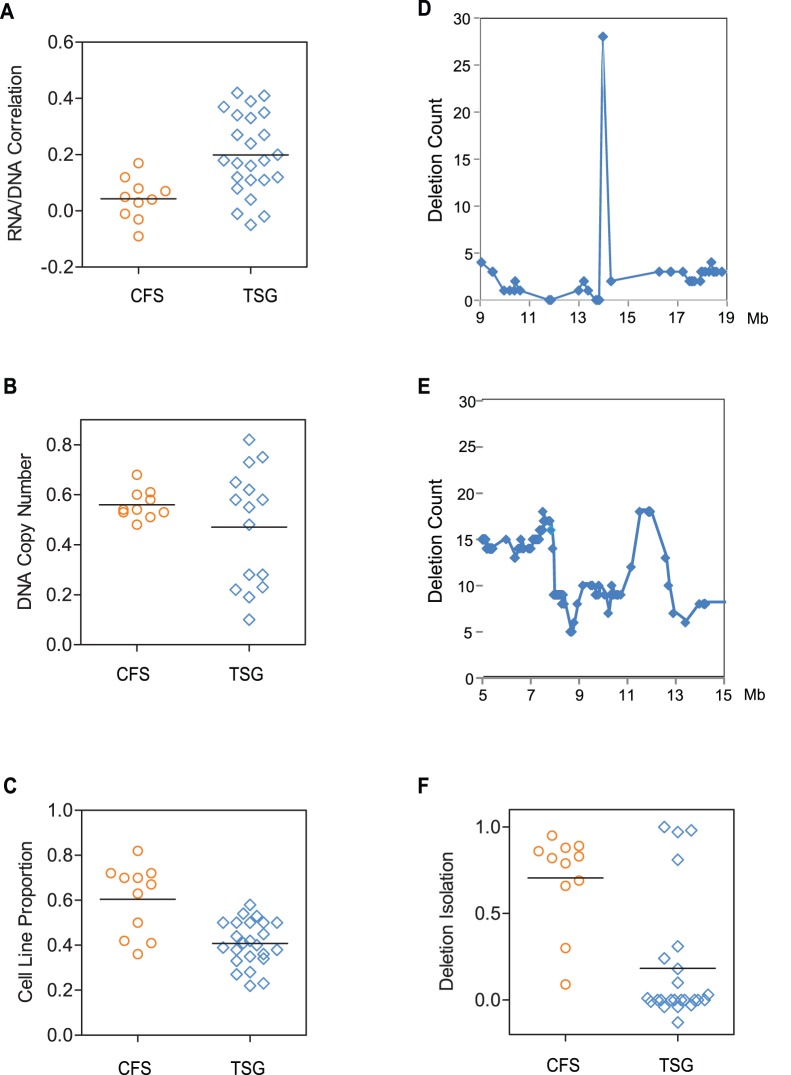
Deletion properties that distinguish common fragile site genes from tumor suppressor genes. Scatter dot plots of values for statistics that distinguish focal deletions affecting common fragile site (CFS) genes (orange) from tumor suppressor genes (blue). (A) “RNA/DNA Correlation,” which corresponds to the Pearson’s correlation coefficient of log2-transformed DNA copy number values and relative RNA expression values for the 115 tumor samples with both array CGH and RNA expression profiling data, (B) DNA Copy Number is the median value of segmented DNA copy number values. (C) Cell Line Proportion” which provides a relative measure of how frequently deletions for a given gene are found in cell lines rather than primary tumors. Panels (D) and (E) show the number of deletions found in each gene within a 10 Mb region centered on the *MACROD2* locus (D) and TP53 and *MAP2K4* loci (E). (F) Box and whisker plots of “Deletion Isolation,” which measures how frequently neighboring genes are deleted relative to the featured gene.

We next wanted to determine whether the inability of deletions to affect expression of *MACROD2* was a more generalizable observation that could be used to distinguish common fragile site-like genes from tumor suppressor genes. We determined the correlation of RNA expression and DNA copy number for 115 cancer samples where we had both gene expression profiling and ROMA aCGH data. Compared to the correlations of the tumor suppressor group, there was very little effect of DNA copy number on gene expression in the common fragile site group (p = .003, Figure 5A), indicating that this feature could be useful in predicting whether or not a given site of deletions was CFS-like. We then examined whether the average DNA copy number value was significantly different, which reflects the degree to which deletions were homozygous versus heterozygous. Even though there wasn’t a statistically significant difference between the average DNA copy number values (p = .09), there did appear to be greater range of lower values for the tumor suppressor group, indicating that some of these genes have more homozygous deletions than do common fragile site genes (Figure 5B).

We also found that deletions affecting common fragile sites were more common in cancer cell lines than in primary tumors when compared to deletions affecting tumor suppressors (p = 7e-05, Figure 5C). This is particularly evident in breast cancer, where *MACROD2* and *FHIT* are deleted in 28% and 15% of breast cancer cell lines, respectively, but either not at all (*FHIT*) or in only one out of 255 primary tumors (*MACROD2*) (Table S5). *FHIT* was co-deleted with *MACROD2* in 75% of the cell lines with *FHIT* deletion, indicating that for certain breast cancer cell lines, replicative stress followed by DNA breakage and repair might occur during adaptation to cell culture and affect multiple common fragile site genes.

By looking at genome coordinate plots of focal deletion frequencies, we noted that the frequent deletions affecting *MACROD2* were relatively isolated along the genome and that the frequency count fell precipitously to the genes immediately left and right of *MACROD2* (Figure 5D). This was very distinct from the genome neighborhood of focal deletions affecting the tumor suppressor genes *TP53* and *MAP2K4*, which both formed peaks of deletion counts but less dramatically in the context of genomic regions with a higher overall rate of deletions (Figure 5E). We wanted to determine whether this distinction was a generalizable feature that distinguished common fragile site genes from tumor suppressor genes, and developed a metric “Deletion Isolation” that measured how much the deletion frequency of a given gene was greater than neighboring genes. This metric was considerably higher in common site fragile genes than tumor suppressor genes (>40-fold, p = 0.00001) (Figure 5F).

### Computational Analysis and Classification of Focal Deletions

**Figure 6:**
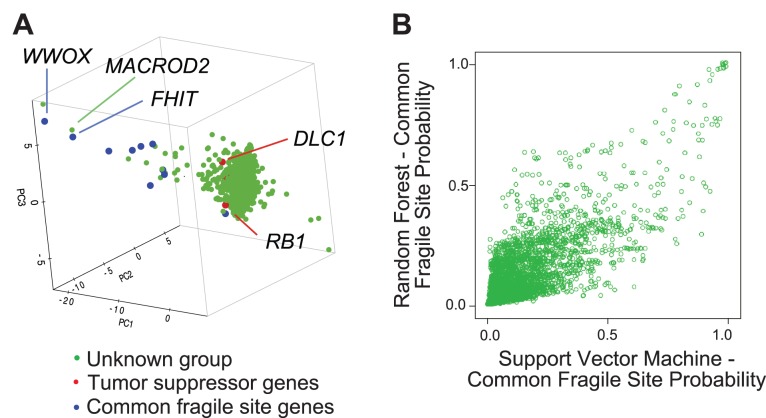
Genome-wide analysis and classification of focal deletions in cancer. Genes that had 15 or more focal deletions (4,823) were analyzed by the unsupervised learning method of Principal Component Analysis (PCA) and classified by two supervised learning methods, Support Vector Machine (SVM) and Random Forest (RF). (A) Graph of the 4,826 genes based on their values for the first three principal components derived from the seven deletion properties that distinguish common fragile site genes from tumor suppressor genes. Known common fragile site genes are shown in blue, known tumor suppressors are shown in red, and all others are shown in green. Most known tumor suppressors are buried within the major group of genes, whereas the known common fragile site genes, with one exception (*LARGE*), are clearly separated from the major group of genes by their first and second principal component values. There are only a handful of other genes that are located near the known common fragile site genes. (B) Graphical representation of the classification of the 4,826 genes based on their probability of being common fragile site genes by Random Forest (*y*-axis) or Support Vector Machine (*x*-axis) classifiers. Both classifiers were trained on the 35 known CFS genes or tumor suppressors (Table S4). The probability of being a tumor suppressor is one minus the CFS probability. Most genes are clustered together in the lower left quadrant; therefore, both classifiers predict with high probability that most of these genes are tumor suppressors.

We wanted to use these six properties to analyze deletions on a genome-wide scale. We restricted our attention to genes with sufficient deletions in order to obtain statistically meaningful results (>14 deletions; 4,823 genes). We first explored whether any of the six properties were redundant by determining if they were highly correlated with any of the other five. Although deletion size was moderately correlated with deletion separation (r = 0.39), all of the other pairwise correlations were negligible (ranging from −0.13 to 0.18), and we proceeded to include all six properties for unsupervised analysis. We then transformed the six properties using principal component analysis and plotted the 4,823 genes using the first three principal components. The resultant graph showed that the majority of genes were most similar to recessive tumor suppressor genes (Figure 6A). With one exception, common fragile site genes were well separated from the main group and were similar to only a small number of additional genes (Figure 6A). This result indicated that most genes that are targeted by focal deletions are similar to tumor suppressor genes, whereas considerably fewer genes are similar to common fragile site genes. To test this idea independently, we used supervised learning methods (support vector machine and random forest [RF]) to classify the 4,823 genes into CFS-like or tumor suppressor categories. The results of these two methods were significantly correlated (r = 0.72) and both classified most genes as tumor suppressor like, in agreement with the unsupervised analysis (Figure 5B). The three variables that were most important in the RF classification were deletion size, deletion separation, and deletion isolation (Table S6). Using just these three variables, we generated a new RF classifier that yielded results that were 99% identical with the original classifier, establishing these three properties as the most critical determinants as to whether a given pattern of focal deletions is CFS-like or tumor suppressor-like.

### Validation of *MACROD2* as a Common Fragile Site Gene

**Figure 7:**
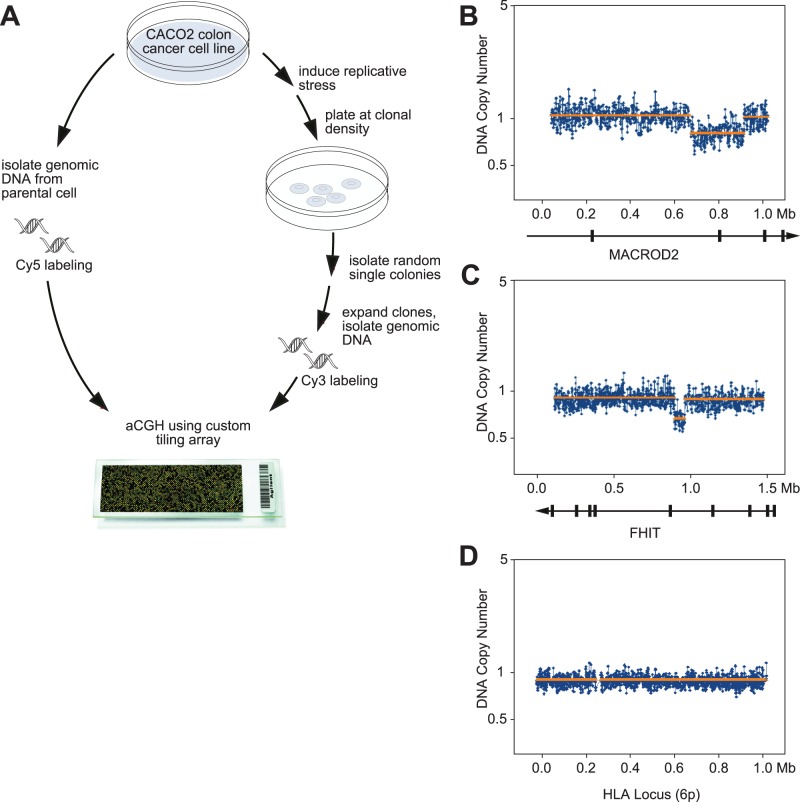
Validation of *MACROD2* as a common fragile site gene. (A) A schematic of the experiment, which used a custom tiling array to determine if aphidicolin induction of replicative stress could generate focal deletions at three loci (*A2BP1*, *MACROD2*, and *FHIT*) in colon epithelial cancer cells. No deletions affecting *A2BP1* were observed in the 8 examined clones; 4 out of 8 clones had deletions affecting *MACROD2*; and 2 clones had deletions affecting *FHIT*. (B) An example of an induced focal deletion affecting the *MACROD2* locus. The *x*-axis represents an approximately 1-Mb region spanning *MACROD2*. The blue lines represent the normalized DNA copy number values, and the orange lines represent the segmented DNA copy number values. (C) An example of an induced focal deletion affecting *FHIT*. (D) An example of an unaffected locus (the HLA locus on chromosome 6p).

To test whether our classification could successfully predict new common fragile site gene genes, we examined whether induction of replicative stress in a colon cancer cell line can generate deletions in *MACROD2* or *A2BP1*, which, together with the known common fragile site gene gene *FHIT,* are the three genes most frequently affected by focal deletions in colon cancer. We designed a custom tiling array for these three genes and then performed aCGH, comparing the original cell line DNA to DNA from eight different clones isolated after brief induction of replicative stress. Four out of eight clones showed deletions within *MACROD2*, and two out of eight clones showed deletions within *FHIT* (Figure 7). This establishes that *MACROD2*, which was not previously shown to be a common fragile site gene in lymphocytes, is indeed a common fragile site gene when assayed in a colon cancer cell line. The fact that *MACROD2* was readily detected as a common fragile site gene in colon cancer cells but not in lymphocytes is consistent with prior evidence that different cell types show different profiles of common fragile sites [24].

### Different Tumor Types have Different Frequencies and Spectrums of Deletions within CFS-like Genes

**Figure 8:**
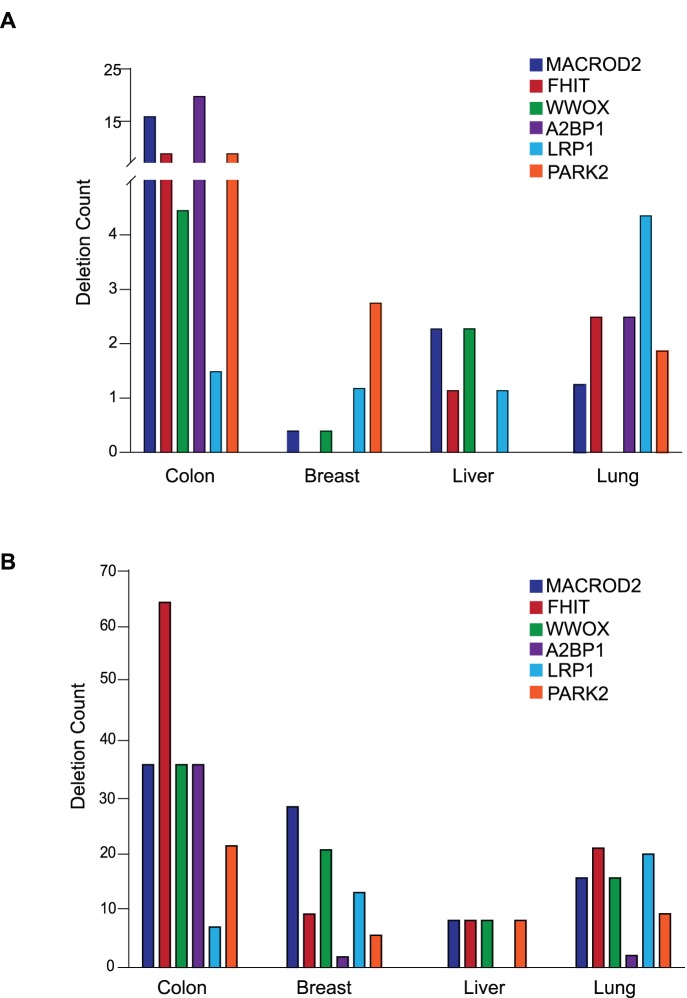
Deletion frequency of CFS-like genes in different tumor types and cancer cell lines. (A) The frequency with which the indicated common fragile site or CFS-like gene is deleted in different primary tumors. (B) The frequency with which the indicated common fragile site or CFS-like gene is deleted in cancer cell lines.

Interestingly, we found that colon tumors were by far the most frequently affected by focal deletions in CFS-like genes, with deletion frequencies as high as 21% for *A2BP1*, 17% for *MACROD2*, 9% for *FHIT* and 9% for *PARK2* (Figure 8A). None of the other nine tumor types were as frequently affected. Lung cancer was the next most affected, but curiously had a different spectrum of frequencies, with *LRP1B* being the most frequently deleted CFS-like gene at 4% (Figure 8A). Several cancers did not appear to be affected at all, including glioblastomas, CLL, and prostate cancers, and many had very low frequency of deletions of CFS-like genes, including breast cancer, which showed *PARK2* as its most frequently deleted CFS-like gene at just above 2% (Figure 8A).

The frequency and spectrum of deletions in CFS-like genes was different for all tumor types when comparing cell lines to primary tumors (Figure 8B). In colon cancer cell lines, deletions affected *FHIT* in over 60% of the cell lines compared to 9% of primary tumors. In breast cancer cell lines, *FHIT* was deleted in 10% of the samples but not at all in primary tumors (Figure 8B). It seems possible that this may reflect the plasticity of the genome at *FRA3B* (the *FHIT* fragile site) under culture conditions, and the same may hold true for the increased incidence of deletions in other CFS-like genes when comparing cell lines to primary tumors.

### The Most Frequently Deleted Fragile Site-like Genes and Tumor Suppressor-like Genes

The ability to predict new tumor suppressors is more pertinent to cancer biology than the ability to predict new fragile site genes. We reasoned that those tumor suppressor-like genes most frequently affected by focal deletions would be amongst the strongest candidates (Table 1). Interestingly, focal deletions in only two of the top ten genes have been previously described (*CDKN2A/B* and *MAP2K4* [25]). Of the other eight genes, one is a tumor suppressor that is known to be inactivated by other genetic mechanisms [26] (*MEN1*), and four are candidate tumor suppressors based on either mutational analysis (*CSMD1* [27]), functional analysis (*CDKN2AIP/CARF* [28], *MAD1L1* [29]), or cancer-specific promoter hypermethylation (*RRAD* [30]). On the other hand, two genes recently proposed to be tumor suppressors based on their focal deletion in cancer, *PDE4D* [31] and *LRP1B* [32], are amongst the top ten most frequently affected CFS-like genes and accordingly may not be functional tumor suppressors (Table 1).

**Table 1:** Predicted and validated fragile site genes and tumor suppressor genes ranked by focal deletion frequency.

	Tumor Suppressor Gene Probability					
Gene Symbol	Random Forest	Support Vector Machine	Focal Deletion Frequency^c^	Deletion Depth^d^	Chromosomal Location	Function^e^
**Tumor suppressor genes:**						
*CDKN2AIP/CARF* a	0.78	0.96	8%	0.73	4q35.1	p14ARF/p53
*CDKN2A/B*	0.96	0.96	8%	0.28	9p21.3	Cdk4/Rb1/p14ARF/p53
*MAD1L1* a	0.97	0.95	7%	0.79	7p22.3-p22.2	Mitotic checkpoint
*CSMD1* a	0.99	0.81	5%	0.41	8p23.2	CUB and Sushi multiple domains
*PARK7* a	0.86	0.98	4%	0.71	1p36.23	Redox-sensitive chaperone
*ARHGAP8* a	0.93	0.87	4%	0.64	22q13.31	Rho-GTPase activating protein
*UNC84A* a	0.93	0.80	3%	0.65	7p22.3	Nuclear envelope protein
*RRAD* a	0.99	0.97	3%	0.68	16q22.1	Ras-related; associated with diabetes
*MEN1*	0.99	0.98	3%	0.73	11q13.1	Transcription factor
*MAP2K4*	0.99	0.91	3%	0.22	17p12	JNK and p38 MAP kinase signaling
**Fragile site genes:**						
*FHIT*	0.00	0.01	7%	0.54	3p14.2	Dinucleoside hydrolase
*MACROD2* ^b^	0.01	0.02	7%	0.59	20p12.1	Macro domains
*A2BP1* ^b^	0.05	0.06	5%	0.54	16p13.2	Alternative splicing factor
*WWOX*	0.02	0.02	5%	0.60	16q23.1	Oxidoreductase
*LRP1B*	0.03	0.04	4%	0.48	2q22.2-q22.1	LDL receptor-related
*PARK2*	0.07	0.07	4%	0.53	6q26	E3 ligase
*PDE4D* ^b^	0.00	0.02	3%	0.51	5q11.2-q12.1	Phosphodiesterase
*FAM190A*	0.00	0.01	2%	0.54	4q22.1	Unknown
*CNTNAP2*	0.02	0.03	2%	0.51	17q35-q36.1	Contactin-associated protein
*DLG2*	0.03	0.08	2%	0.61	11q14.1	Membrane-associated guanylate cyclase

a predicted tumor suppressor gene ^b^predicted common fragile site gene.

c for deletions less than 10 Mb; does not include larger deletions.

d deletions in *CDKN2A/B*, *CSMD1*, and *MAP2K4* tend to be homozygous more often than deletions in other genes.

e functions were assigned from the literature.

## Discussion

We initiated this study to reconcile two disparate findings: the absence of tumor suppressor candidates from two loci frequently affected by focal deletions, and the ability of focal deletions to enrich for tumor suppressors in a functional screen. Through computational and statistical analysis of focal deletions present in more than 1000 cancer samples, we defined two distinct classes of deletions in cancer that resolve this incongruity: a class that represents genes similar to common fragile site genes, and another that is tumor suppressor-like. These two types of deletions are likely to arise from different mechanisms, based on their different deletion sizes and their tendency to co-occur. The one class of deletions, when highly recurrent, overlap a common site and significantly affect the expression of the underlying genes, indicating that recurrence is driven by selective advantage to the evolving tumor cells. In contrast, deletions in common fragile site-like genes do not overlap a common site nor do they have significant effects on underlying gene expression, all of which is consistent with their recurrence being driven by the inherent instability of the genomic locus rather than by selective advantage.

Our findings establish that most focal deletions belong to the class that represents tumor suppressor-like genes and not common fragile site genes. This is the only disagreement we have with a recent study which suggests that most focal deletions in cancer are fragile-like based in part on in part on their propensity to be heterozygous rather than homozygous [16]. However, we believe that this property is not useful as a classifier because several tumor suppressors, including *TP53* and *CDH1*, are affected by focal heterozygous deletions. It may be many haploinsufficient tumor suppressors, such as *p27KIP1* [33], remain to be discovered, and computational tools that discount these would be misguided. In some cases, homozygous deletion of a tumor suppressor might be lethal, which could be the case for the spindle assembly checkpoint gene *MAD1L1* which we found here to be very frequently affected by heterozygous focal deletions. Although *MAD1L1* is not yet in the COSMIC database of tumor suppressors, heterozygous deletion of *MAD1L1* has been shown to increase the incidence of tumors caused by partial loss of *TP53* in mice [29].

One of the most interesting findings of our study was the tumor-type specific pattern of deletions in CFS-like genes. There appears to be a clear distinction in many cases from known common fragile site genes, which have been determined almost exclusively from analysis of DNA breaks in lymphocytes [1]. Indeed, many of the most commonly deleted CFS-like genes in cancer, including *A2BP1*, *MACROD2*, and *PDE4D*, are not known common fragile sites even in the most recent analyses [2]. Although we initially hypothesized that significantly different chromatin structure within different tissue types could underlie at least part of tumor-type specific patterns of deletions in CFS and CFS-like genes, we observed no correlation with chromatin structure as indicated by DNAase I hypersensitivity data from ENCODE (Table S9). We think a more likely explanation for the tissue-type diversity comes from the findings that both replication origin setting and replication timing are tissue specific in mammalian cells and that this explains the diversity of common site breakage at the *FRA3B* locus [34].

From the six deletion properties that could be used to distinguish CFS-like genes, machine learning analysis with the random forests classifier determined that only three deletion properties are necessary to classify genes as being CFS-like or not: deletion size, deletion separation, and deletion isolation. These properties should be useful for investigators interested in determining if their particular deleted candidate tumor suppressor gene is CFS-like or not.

## Methods

### Online Access to Microarray Data

We have deposited the aCGH microarray data with the Gene Expression Omnibus (GEO) repository, accessions GSE31586 and GSE22916.

### Tumor Samples, Xenografts, and Cell Lines

We analyzed 850 tumor samples for this study (Table S1). Many of them have been previously described, including the 255 breast cancer samples from the Cancer Center of the Karolinska Institute, Sweden, and the Oslo University Hospital, Norway [35]; the 161 lung cancer samples from the Cooperative Human Tissue Network (CHTN) [9]; the 88 liver cancer samples from CHTN and medical centers in Germany and Hong Kong [15]; and the 27 pancreatic cancer samples and 40 pancreatic cancer xenografts from Johns Hopkins University and the Arizona Cancer Center [36]. New to this study are samples that were obtained after approval from their respective institutional review boards as indicated below. All patients provided written informed consent and samples were procured under the respective Institutional Review Board approvals. These include 135 colon cancer samples which were obtained from Case Western Reserve University collected under an Institutional Review Board (IRB) approved protocol at the Case Medical Center; 106 ovarian cancer samples which were obtained from Memorial Sloan Kettering Cancer Center (MSKCC) under a protocol approved by the MSKCC Human Biospecimen Utilization Committee, a Norwegian cohort from the Oslo University Hospital approved by the Regional Committees for Medical and Health Research Ethics (REC) board, the University of Michigan with a protocol approved by the Institutional Review Boards of the University of Michigan Medical School (IRBMED); 29 melanoma samples which were obtained from MSKCC under a protocol approved by the Memorial Hospital Institutional Review Board; 27 prostate cancer samples which were obtained under clinical research approvals from the IRB of the Karolinska Hospital; and 22 glioblastoma samples which were obtained from the Brain Tumor and Neuro-Oncology Center under approval of the Institutional Review Board of the Neurological Institute of the Cleveland Clinic. This new data has been deposited with NCBI GEO Datasets (GSE31586). DNA and RNA were extracted from dissected tissue containing greater than 75% tumor cell content. The 264 cell lines were obtained from either the American Type Culture Collection (ATCC), the Deutsche Sammlung von Mikroorganismen und Zellkulturen (DSMZ), or the Japanese Collection of Research Bioresources (JCRB).

### Array CGH Analysis

200 ng of genomic DNA was used to make representations for whole-genome copy number analysis by ROMA array comparative genomic hybridization as described [35]. The probes were mapped to the March 2006 human reference sequence (NCBI Build 36.1). Hybridizations, washing, scanning, and data normalization were performed as described [35]. The majority of cancer samples were co-hybridized to the arrays with a differentially-labeled unrelated reference genome, although in some cases matching normal DNA was used as the reference. The normalized fluorescent ratios representing DNA copy number measurements were segmented using the CBS segmenter [37].

### Identification and Validation of Focal Deletions

Common germline copy number polymorphisms (>5% frequency) were removed from the CBS segmented data by previously described methods [35] using a masking dataset of germline DNA copy number profiles from 500 different normal individuals analyzed with the same platform and CBS segmentation algorithm. Thresholds for focal deletions included a minimum of three probes per segment, a segmented DNA copy number value that was 0.85 or below and at least 0.1 lower than both nearest neighboring segments, and a segment size of less than 10 Mb. We validated this approach to aCGH-based determination of focal deletions by confirming the presence and boundaries of deletions by real-time PCR using nine different TaqMan probes (Table S7).

### Deletion Metrics and Classification of Focal Deletions

The set of 10,835 focal deletions was used to develop metrics that were based on 2 Mb intervals (windows). For genes that were affected by 8 or more deletions, we constructed a set of windows that contained deletions based on the deletion start and stop positions, and assigned to the gene the values of the closest possible window (center of gene to center of the window). “Deletion Size” is the median length of all deletions that overlap the window. “Deletion Separation” is calculated by determining for each deletion how many other deletions in the window are separate (non-overlapping), then taking the average of these values, and then standardizing this score so that is independent of the number of deletions.

Other metrics were calculated on the level of individual genes. “Deletion Isolation” was developed to quantify whether deletions also affected nearby genes. For each gene, a gene-size neutral metric was determined by summing the fraction of the gene deleted in all samples. Deletion Isolation was then determined by subtracting the average of the same metric for both the nearest upstream and nearest downstream neighboring genes. “Cell Line Proportion” was determined from the fraction of deletions in a given gene that occurred in cell lines relative to the total number of deletions in all sample types. “RNA/DNA Correlation” for each gene was calculated from the Pearson correlation coefficient of the log2-transformed segmented DNA copy number value with the log2-transformed relative RNA expression level. For genes with more than one probe on the Nimblegen expression array (see Supplementary Methods), the average value was used.

### PCA and Machine Learning Tools

Principal Component Analyasis was performed for the 4,859 genes using the mean-centered and variance-adjusted seven parameters with a built-in function from the ‘labDSV’ R software package. We used two classifiers, Random Forest (RF) and Support Vector Machine (SVM), to perform genome-wide classification of deleted genes with respect to their tumor suppressor-like or CFS-like properties. We used the Random Forest algorithm as implemented in the randomForest R software package (http://www.R-project.org). The algorithm was trained on a set of 24 known tumor suppressors and 11 known common fragile site genes (Table S4). We grew 10,000 trees each time we generated a classifier. Initially all seven predictor variables were used for training, resulting in correct out-of-bag classification of all tumor suppressors and all but two common fragile site genes. The importance of individual predictors was measured by mean decrease of the Gini index. We then trained the classifier again, retaining the three most important predictors: “Deletion Separation”, “Deletion Size” and “Deletion Isolation”. The quality of out-of-bag classification was identical to that with the full set of predictors. Finally, we applied both classifiers to the entire set of 4,823 genes focally deleted in more than 14 tumors each. The predicted class (tumor suppressor or CFS-like) differed for only 39 genes between the two RF classifiers (Table S8). For SVM classification, we used the Kernel-based Machine Learning Lab R software package (http://cran.r-project.org/). We used the Gaussian kernel, and set the kernel function parameter to 0.01 and the soft margin parameter *C* to 10, based on a grid search for the best (three-fold) cross-validation error. The final cross-validation error was 3.2% and the training error was 2.9%. To assign class probabilities, we used the ‘prob.model’ option (Table S8).

### Co-deletion Values

The co-deletion values were calculated by first constructing a deletion vector for each of the 35 genes. Supposing there were only 5 samples, and gene A is deleted in sample 1 and sample 3, and gene B is deleted in sample 2 and sample 3, then the co-deletion value is the correlation between (1, 0, 1, 0, 0) and (0, 1, 1, 0, 0). Only samples that contained at least 1 deletion in the set of 35 genes were used in this analysis.

### Expression Analysis

For a subset of the cancer samples, we performed whole-genome expression profiling using Nimblegen’s recommended single-color hybridization protocol and their 385,000 probe gene expression array (design ID #1877). Gene calls were generated using the Robust Multichip Average (RMA) algorithm provided with the NimbleScan software package. Subsequently, RMA expression values for a given tumor type were quantile normalized using R. Before computing the correlation between DNA copy number and expression, the normalized RMA expression values were log2-transformed and standardized with values obtained with Stratagene’s universal reference RNA.

### Biological Assays

Induction of focal deletions by replicative stress was performed by treatment of Caco-2 cells with a subtoxic dose (0.3 µM) of aphidicolin (Calbiochem) for 5 days followed by 24 hours recovery. Treated cells were trypsinized and 150 cells were plated onto 10 cm plates. After two weeks, clones were selected and expanded. Genomic DNA was isolated from each clone using the Gentra Puregene Core kit (Qiagen) and used to generate probes for array CGH analysis. Microarrays were custom designed to tile the *MACROD2*, *A2BP1*, and *FHIT* loci with Agilent’s eArray tool (https://earray.chem.agilent.com/earray) using an 8×15K format. Arrays were hybridized and scanned as recommended by the manufacturer and following normalization the fluorescent ratios representing DNA copy number measurements segmented using the CBS segmenter.

Taqman probes were designed with ABI software and used as described for both real-time PCR analyais of DNA and RNA [10]. We constructed two *A2BP1* expression vectors (pMSCV-A2BP1 and pMSCV-FlagA2BP1) by using high-fidelity PCR to amplify the coding-sequence insert from cDNA clone AF107203.1 (alternate splicing isoform 3) with primers encoding a wild-type N-terminus (for pMSCV-A2BP1) or using the insert from cDNA clone NM_018723.2 (alternate splicing isoform 4) using primers encoding a Flag-tagged N-terminus. We chose these two isoforms for functional studies as they were the most abundant based on annotation at http://genome.ucsc.edu. After validation by DNA sequencing, these plasmids were transfected into cancer cell lines and assayed for effects on tumorigenicity in nude mice as described [10]. The tumorigenicity studies were approved by CSHL’s Institutional Animal Care and Use Committee (IACUC).

## Supporting Information

Figure S1 **Average DNA segment sizes.** Plotted as a function of position on chromosomes 2 through 13.
(EPS)

Table S1 **Tumor samples, cancer cell lines, and xenografts used in this study.** This table contains four columns describing for each sample the tumor type, sample ID, array CGH ID, and sample type.
(XLS)

Table S2 **Properties of the 10,835 focal deletions.** This table contains ten columns describing for each focal deletion the tumor type, chromosome (23 = X), chromosomal start position (hg18), chromosomal stop position, the start and stop position on an absolute genome scale, the segmented DNA copy number value, the array CGH ID, and the sample type.
(XLS)

Table S3 **Number of focal deletion involving individual telomeres.** Focal deletions (out of the total of 10,835) that effect individual telomeres are shown.
(XLSX)

Table S4 **The known common fragile site genes and tumor suppressor genes affected by ≥15 focal deletions.** This set of genes was used for exploring properties that distinguished the two classes and for the training sets for the machine-learning tools.
(XLS)

Table S5 **Differential frequency of deletions in CFS genes between breast cancer cell lines and primary breast tumors.** This table show the numbers of breast cancer samples affected by focal deletions in the indicated genes, broken out by sample type (tumor or cell line). The average number of deletions affecting RefSeq genes is shown in the last row.
(XLSX)

Table S6 **The importance of individual predictors in the RF classifier.** The mean decrease in the Gini index was used to calculate the relative important of the seven individual properties in the RF classifier.
(XLSX)

Table S7 **Validation of focal deletion calls by TaqMan real-time PCR.** TaqMan probes were designed to nine different locations within the A2BP1 locus and used to determine DNA copy number in four different colon cancer cell lines. Shown for each cell line are two rows comparing the DNA copy number estimates based on aCGH versus real-time PCR. Note that there is good agreement except for the boundary of the hemizygous deletion in the cell line LIM2045. Real-time PCR has a greater dynamic range and can more accurately distinguish homozygous from heterozygous deletions than aCGH.
(XLSX)

Table S8 **Functional classification of 4,823 genes for their TSG or CFS probabilities.** This table contains fourteen columns describing for each gene with >15 focal deletions the chromosome, chromosomal start position, chromosomal stop position, the seven properties that distinguish TSG from CFS genes (mean-centered and variance-adjusted), the probability of being a TSG based on Random Forest classification (using either 7 or 3 properties), and the probability of being a TSG based on SVM classification. The probability of being a CFS is 1– TSG probability.
(XLS)

Table S9 **DNAse I hypersensitivity for six common fragile site or CFS-like genes in four different cell types.** The average DNAse I hypersensitivity for the indicated genes was taken from ENCODE data from the University of Washington (http://genome.ucsc.edu/cgi-bin/hgFileUi?db=hg19&g=wgEncodeUwDnase). Higher numbers indicate greater sensitivity and more open chromatin structure.
(DOCX)

## References

[1] Durkin SG, Glover TW (2007) Chromosome fragile sites. Annu Rev Genet 41: 169–192. 17608616 10.1146/annurev.genet.41.042007.165900

[2] Fungtammasan A, Walsh E, Chiaromonte F, Eckert KA, Makova KD (2012) A genome-wide analysis of common fragile sites: what features determine chromosomal instability in the human genome? Genome Res 22: 993–1005. 22456607 10.1101/gr.134395.111 PMC3371707

[3] Huebner K, Croce CM (2001) FRA3B and other common fragile sites: the weakest links. Nat Rev Cancer 1: 214–221. 11902576 10.1038/35106058

[4] Abdeen SK, Salah Z, Maly B, Smith Y, Tufail R, et al (2011) Wwox inactivation enhances mammary tumorigenesis. Oncogene 30: 3900–3906. 21499303 10.1038/onc.2011.115

[5] Poulogiannis G, McIntyre RE, Dimitriadi M, Apps JR, Wilson CH, et al (2010) PARK2 deletions occur frequently in sporadic colorectal cancer and accelerate adenoma development in Apc mutant mice. Proc Natl Acad Sci U S A 107: 15145–15150. 20696900 10.1073/pnas.1009941107 PMC2930574

[6] Je EM, Yoo NJ, Lee SH (2012) Somatic Mutation of PARK2 Tumor Suppressor Gene is not Common in Common Solid Cancers. Pathol Oncol Res. 10.1007/s12253-012-9591-z 23225159

[7] Thiagalingam S, Lisitsyn NA, Hamaguchi M, Wigler MH, Willson JK, et al (1996) Evaluation of the FHIT gene in colorectal cancers. Cancer Res 56: 2936–2939. 8674044

[8] Watanabe A, Hippo Y, Taniguchi H, Iwanari H, Yashiro M, et al (2003) An opposing view on WWOX protein function as a tumor suppressor. Cancer Res 63: 8629–8633. 14695174

[9] Kendall J, Liu Q, Bakleh A, Krasnitz A, Nguyen KC, et al (2007) Oncogenic cooperation and coamplification of developmental transcription factor genes in lung cancer. Proc Natl Acad Sci U S A 104: 16663–16668. 17925434 10.1073/pnas.0708286104 PMC2034240

[10] Mu D, Chen L, Zhang X, See LH, Koch CM, et al (2003) Genomic amplification and oncogenic properties of the KCNK9 potassium channel gene. Cancer Cell 3: 297–302. 12676587 10.1016/s1535-6108(03)00054-0

[11] Zender L, Spector MS, Xue W, Flemming P, Cordon-Cardo C, et al (2006) Identification and validation of oncogenes in liver cancer using an integrative oncogenomic approach. Cell 125: 1253–1267. 16814713 10.1016/j.cell.2006.05.030 PMC3026384

[12] Li J, Yang Y, Peng Y, Austin RJ, van Eyndhoven WG, et al (2002) Oncogenic properties of PPM1D located within a breast cancer amplification epicenter at 17q23. Nat Genet 31: 133–134. 12021784 10.1038/ng888

[13] van der Horst EH, Degenhardt YY, Strelow A, Slavin A, Chinn L, et al (2005) Metastatic properties and genomic amplification of the tyrosine kinase gene ACK1. Proc Natl Acad Sci U S A 102: 15901–15906. 16247015 10.1073/pnas.0508014102 PMC1276100

[14] Sawey ET, Chanrion M, Cai C, Wu G, Zhang J, et al (2011) Identification of a therapeutic strategy targeting amplified FGF19 in liver cancer by Oncogenomic screening. Cancer Cell 19: 347–358. 21397858 10.1016/j.ccr.2011.01.040 PMC3061399

[15] Zender L, Xue W, Zuber J, Semighini CP, Krasnitz A, et al (2008) An oncogenomics-based in vivo RNAi screen identifies tumor suppressors in liver cancer. Cell 135: 852–864. 19012953 10.1016/j.cell.2008.09.061 PMC2990916

[16] Bignell GR, Greenman CD, Davies H, Butler AP, Edkins S, et al (2010) Signatures of mutation and selection in the cancer genome. Nature 463: 893–898. 20164919 10.1038/nature08768 PMC3145113

[17] Cancer Genome Atlast Network (2012) Comprehensive molecular characterization of human colon and rectal cancer. Nature 487: 330–337. 22810696 10.1038/nature11252 PMC3401966

[18] Andersen CL, Lamy P, Thorsen K, Kjeldsen E, Wikman F, et al (2011) Frequent genomic loss at chr16p13.2 is associated with poor prognosis in colorectal cancer. Int J Cancer 129: 1848–1858. 21154748 10.1002/ijc.25841

[19] Kuroyanagi H (2009) Fox-1 family of RNA-binding proteins. Cell Mol Life Sci 66: 3895–3907. 19688295 10.1007/s00018-009-0120-5 PMC2777236

[20] Forbes SA, Tang G, Bindal N, Bamford S, Dawson E, et al (2010) COSMIC (the Catalogue of Somatic Mutations in Cancer): a resource to investigate acquired mutations in human cancer. Nucleic Acids Res 38: D652–657. 19906727 10.1093/nar/gkp995 PMC2808858

[21] McAvoy S, Ganapathiraju SC, Ducharme-Smith AL, Pritchett JR, Kosari F, et al (2007) Non-random inactivation of large common fragile site genes in different cancers. Cytogenet Genome Res 118: 260–269. 18000379 10.1159/000108309

[22] Arlt MF, Mulle JG, Schaibley VM, Ragland RL, Durkin SG, et al (2009) Replication stress induces genome-wide copy number changes in human cells that resemble polymorphic and pathogenic variants. Am J Hum Genet 84: 339–350. 19232554 10.1016/j.ajhg.2009.01.024 PMC2667984

[23] Raschke S, Balz V, Efferth T, Schulz WA, Florl AR (2005) Homozygous deletions of CDKN2A caused by alternative mechanisms in various human cancer cell lines. Genes Chromosomes Cancer 42: 58–67. 15495191 10.1002/gcc.20119

[24] Murano I, Kuwano A, Kajii T (1989) Fibroblast-specific common fragile sites induced by aphidicolin. Hum Genet 83: 45–48. 2504659 10.1007/BF00274145

[25] Teng DH, Perry WL 3rd, Hogan JK, Baumgard M, Bell R, et al (1997) Human mitogen-activated protein kinase kinase 4 as a candidate tumor suppressor. Cancer Res 57: 4177–4182. 9331070

[26] Agarwal SK, Kester MB, Debelenko LV, Heppner C, Emmert-Buck MR, et al (1997) Germline mutations of the MEN1 gene in familial multiple endocrine neoplasia type 1 and related states. Hum Mol Genet 6: 1169–1175. 9215689 10.1093/hmg/6.7.1169

[27] Farrell C, Crimm H, Meeh P, Croshaw R, Barbar T, et al (2008) Somatic mutations to CSMD1 in colorectal adenocarcinomas. Cancer Biol Ther 7: 609–613. 18614856 10.4161/cbt.7.4.5623

[28] Hasan K, Cheung C, Kaul Z, Shah N, Sakaushi S, et al (2009) CARF Is a vital dual regulator of cellular senescence and apoptosis. J Biol Chem 284: 1664–1672. 19001376 10.1074/jbc.M805778200

[29] Chi YH, Ward JM, Cheng LI, Yasunaga J, Jeang KT (2009) Spindle assembly checkpoint and p53 deficiencies cooperate for tumorigenesis in mice. Int J Cancer 124: 1483–1489. 19065665 10.1002/ijc.24094 PMC2706662

[30] Suzuki M, Toyooka S, Shivapurkar N, Shigematsu H, Miyajima K, et al (2005) Aberrant methylation profile of human malignant mesotheliomas and its relationship to SV40 infection. Oncogene 24: 1302–1308. 15592515 10.1038/sj.onc.1208263

[31] Nancarrow DJ, Handoko HY, Smithers BM, Gotley DC, Drew PA, et al (2008) Genome-wide copy number analysis in esophageal adenocarcinoma using high-density single-nucleotide polymorphism arrays. Cancer Res 68: 4163–4172. 18519675 10.1158/0008-5472.CAN-07-6710

[32] Ding L, Getz G, Wheeler DA, Mardis ER, McLellan MD, et al (2008) Somatic mutations affect key pathways in lung adenocarcinoma. Nature 455: 1069–1075. 18948947 10.1038/nature07423 PMC2694412

[33] Komuro H, Valentine MB, Rubnitz JE, Saito M, Raimondi SC, et al (1999) p27KIP1 deletions in childhood acute lymphoblastic leukemia. Neoplasia 1: 253–261. 10935480 10.1038/sj.neo.7900033 PMC1508076

[34] Letessier A, Millot GA, Koundrioukoff S, Lachages AM, Vogt N, et al (2011) Cell-type-specific replication initiation programs set fragility of the FRA3B fragile site. Nature 470: 120–123. 21258320 10.1038/nature09745

[35] Hicks J, Krasnitz A, Lakshmi B, Navin NE, Riggs M, et al (2006) Novel patterns of genome rearrangement and their association with survival in breast cancer. Genome Res 16: 1465–1479. 17142309 10.1101/gr.5460106 PMC1665631

[36] Chen S, Auletta T, Dovirak O, Hutter C, Kuntz K, et al.. (2008) Copy number alterations in pancreatic cancer identify recurrent PAK4 amplification. Cancer Biol Ther 7. 10.4161/cbt.7.11.6840 PMC7323936 18836286

[37] Venkatraman ES, Olshen AB (2007) A faster circular binary segmentation algorithm for the analysis of array CGH data. Bioinformatics 23: 657–663. 17234643 10.1093/bioinformatics/btl646

[38] Zhang C, Zhang Z, Castle J, Sun S, Johnson J, et al (2008) Defining the regulatory network of the tissue-specific splicing factors Fox-1 and Fox-2. Genes Dev 22: 2550–2563. 18794351 10.1101/gad.1703108 PMC2546699

