# Supplementary material for: Two Distinct Categories of Focal Deletions in Cancer Genomes
Source: PLoS One. 2013 Jun 21;8(6):e66264. doi: 10.1371/journal.pone.0066264 (PMC3689739; doi:10.1371/journal.pone.0066264)
Supplement: Table S9 — DNAse I hypersensitivity for six common fragile site or CFS-like genes in four different cell types. The average DNAse I hypersensitivity for the indicated genes was taken from ENCODE data from the University of Washington (http://genome.ucsc.edu/cgi-bin/hgFileUi?db=hg19&g=wgEncodeUwDnase). Higher numbers indicate greater sensitivity and more open chromatin structure. (DOCX) [file pone.0066264.s010.docx]

| Gene | Tissue Type | | | | | | |
| --- | --- | --- | --- | --- | --- | --- | --- |
|  | Lung | Liver | Colon | | Breast | | |
|  | A549 | Hep-G2 | Caco-2 | HCT-116 | HMEC | T47-D | MCF-7 |
| *A2BP1-RBFOX1* | 1.8 | 1.7 | 1.7 | 1.7 | 1.7 | 1.9 | 2.3 |
| *FHIT* | 2.0 | 2.0 | 2.0 | 3.1 | 2.9 | 2.9 | 2.7 |
| *LRP1B* | 2.4 | 1.7 | 1.6 | 2.3 | 3.0 | 2.2 | 3.0 |
| *MACROD2* | 4.7 | 3.2 | 4.8 | 2.5 | 6.0 | 3.2 | 4.8 |
| *PARK2* | 3.1 | 2.1 | 2.1 | 3.3 | 3.8 | 2.8 | 2.7 |
| *WWOX* | 2.6 | 6.1 | 8.5 | 2.8 | 4.8 | 4.4 | 5.0 |
